# Acidic Tumor Microenvironment Promotes Pancreatic Cancer through miR-451a/MEF2D Axis

**DOI:** 10.1155/2022/3966386

**Published:** 2022-01-12

**Authors:** Jingyong Xu, Yao Li, Zhe Li, Weiwei Shao, Jinghai Song, Junmin Wei

**Affiliations:** Department of General Surgery & Hepato-Bilio-Pancreatic Surgery, Beijing Hospital, National Center of Gerontology, Institute of Geriatric Medicine, Chinese Academy of Medical Sciences, Beijing 100730, China

## Abstract

Pancreatic cancer (PC), as a highly malignant and aggressive solid tumor, is common in the digestive system. The acidic microenvironment is one of the critical markers of cancer. Nonetheless, there are few studies on how the acidic microenvironment affects the development of PC. This study focused on investigating the specific molecular mechanisms of the acidic microenvironment in PC. In our study, qRT-PCR was conducted for examining microRNA (miR)-451a and myocyte enhancer factor 2D (MEF2D) expressions in PANC-1 cells. Then, detailed functional effects of an acidic environment on miR-451a and MEF2D in PANC-1 cells were detected by CCK-8, colony formation, flow cytometry, wound healing, transwell, mitochondrial functionality measurement, JC-1 staining, DCFH-DA staining, and sphere formation assays. The relationship between miR-451a and MEF2D was confirmed by luciferase reporter analysis. Under acidic conditions, the increase of proliferation, migration, and invasion of PANC-1 cells was observed. Moreover, the mitochondrial oxidative respiration-related gene miR-451a was reduced in acidic conditions. In addition, we found that, in PANC-1 cells under an acidic environment, miR-451a overexpression enhanced oxygen consumption, mitochondrial membrane potential (MMP) loss, and ROS generation and inhibited proliferation, migration, invasion, and stemness via sponging MEF2D. In a word, our results revealed that the acidic microenvironment regulated PC progression by affecting the miR-451a/MEF2D axis, indicating a novel avenue for the future treatment of PC.

## 1. Introduction

Pancreatic cancer (PC), as the most common malignant solid tumor of the digestive system, is characterized by high malignancy and invasiveness, and poor prognosis, with less than 8% 5-year survival rate, which is one of the greatest challenges in modern oncology research [1–3]. Based on the Global Cancer Statistics report in 2018, the incidence of PC was 2.5%, while the mortality rate was as high as 4.5% [4]. Moreover, PC may become the second most fatal malignancy by 2030 [5]. The high mortality rate of PC is mainly due to its strong ability to invasion and metastasis. Studies show that PC has begun to invade and metastasize in the early stages [6]. Thus, it is essential to explore the molecular mechanisms of PC invasion and metastasis.

Emerging evidence has indicated that the acidic microenvironment is one of the critical markers of the tumor [7, 8]. Studies revealed that most tumor cells exhibit the metabolic adaptability of the “Warburg” effect, that is, regardless of whether there is sufficient oxygen, tumor cells tend to produce energy through glycolysis, which significantly increases glucose metabolism and then produces a large amount of lactic acid [9, 10]. In addition, insufficient perfusion and chaotic vascular structure further increase the accumulation of lactic acid and hydrogen ions in the tumor microenvironment [11]. Therefore, compared with normal tissue, the extracellular environment of tumor tissue is acidic, with a pH range of 5.5 to 7.0 [12]. In the early stage of tumor cell growth, the acidic environment makes the cells more aggressive, allowing cancer cells to invade normal tissues [13]. In addition, the generation of an acidic microenvironment induced with tumors including PC can inhibit tumor cell apoptosis and promote cell proliferation, invasion, and immune escape [7, 14]. Thus, whether the acidic microenvironment contributes to the progression of PC aroused our concern.

MicroRNAs (miRNAs) are single-stranded noncoding RNAs with a length of about 21–23 nt, involved in regulating post-transcriptional gene expression. Through targeting the 3′-UTR region of target genes, miRNAs regulate growth and survival through gene inhibition, thereby inhibiting translation or accelerating mRNA degradation [15]. Currently, miRNAs have been found to possess substantial impacts on cell activities, such as proliferation, differentiation, and apoptosis and are involved in the progression of most malignant tumors, including PC [16, 17].

Interestingly, to get with the acidic hypoxic microenvironment, many miRNAs are abnormally expressed in PC cells [18, 19]. Clarence et al. reported that miR-451a as a tumor suppressor was downregulated in the hypopharyngeal carcinogenesis exposure to weakly acidic bile [20]. Among those dysregulating miRNAs in the acidic microenvironment, miR-451a, as an attractive candidate, was low expressed under acidic conditions. Importantly, myocyte enhancer factor 2D (MEF2D), a member of the MEF2 family exhibiting functional effects on the progression of tumors including PC [21], might be a potential target of miR-451a according to bioinformatics prediction. Therefore, whether miR-451a regulates the proliferation, migration, invasion, and apoptosis of PC cells through MEF2D under an acidic microenvironment was investigated in this study.

## 2. Materials and Methods

### 2.1. Cell Culture

The PC cell line (PANC-1) was provided by ATCC (Manassas, VA, USA). It was cultivated within DMEM containing 10% fetal bovine serum (FBS; Procell, Wuhan, China), 100 U/mL penicillin, and 100 *μ*g/mL streptomycin. Furthermore, the culture medium (pH 6.8) was for acidic conditions and pH 7.4 for normal conditions [7].

### 2.2. Cell Transfection

The miR-451a mimics, sh-RNA for MEF2D (sh-MEF2D), pc-MEF2D, and corresponding relative controls (miR-NC, sh-NC, and pc-NC) were obtained from Ambion (Austin, USA) and transfected into PANC-1 cells (70–80% confluence) maintained within the 6-well plates using Lipofectamine 3000 (Invitrogen, USA) for 48 h at 37°C. Transfection efficiency was determined by qRT-PCR assay [18].

### 2.3. CCK-8 Assay

A CCK-8 assay (Yeasen, Shanghai, China) was performed to evaluate cell proliferation according to the manufacturer's instructions. In brief, PANC-1 cells (3 × 10^3^ cells) were inoculated into 96-well plates. After being incubated at 37°C for 24, 48, and 72 h, subsequently, 10 *µ*L CCK-8 reagent was added to each well and incubated at 37°C for 4 h. This study measured absorbance (OD) at 450 nm using a spectrophotometer (Molecular Device, San Jose, USA) [22].

### 2.4. Western Blot Assay

Protein was isolated from PANC-1 cells and measured through the BCA kit (Beyotime Biotechnology, China). First, the protein was extracted using 12% SDS-PAGE and then shifted into PVDF membranes (Millipore, USA), which were next incubated using 5% skimmed milk, followed by overnight incubation with primary antibodies under 4°C. After rinsing the membranes, they were incubated for 1 h using HRP-labeled secondary antibody (1:4,000, SA00004-10; Proteintech, China) under ambient temperature. Finally, the enhanced chemiluminescence kit (ECL; Millipore, Bedford, USA) was utilized to observe protein blots, whereas ImageJ software (NIH, version 4.3) was adopted for quantification. All primary antibodies used included anti-PCNA (1:2,000, 10205-2-AP; Proteintech, China), anti-Ki-67 (1:2,000, 27309-1-AP; Proteintech, China), anti-MMP-2 (1:2,000, 10373-2-AP; Proteintech, China), anti-MMP-9 (1:2,000, 10375-2-AP; Proteintech, China), anti-MEF2D (1:2,000, 14353-1-AP; Proteintech, China), anti-CD24 (1:2,000, 18330-1-AP; Proteintech, China), anti-CD44 (1:2,000, 15675-1-AP; Proteintech, China), anti-ESA (1:500; Abcam, USA), and anti-*β*-actin (1:5,000, 66009-1-Ig; Proteintech, China), with *β*-actin being the endogenous control.

### 2.5. Colony Formation Assay

Colony formation assay was performed as previously described [23]. PANC-1 cells were cultured within 6-well plates, and the medium was replaced every 2–3 days for a total of two weeks. Subsequently, cells were stained with 0.5% crystal violet for 15 min and imaged via a light microscope (Nikon, Japan).

### 2.6. Flow Cytometry

Apoptosis was detected using flow cytometry. PANC-1 cells were trypsin and rinsed by PBS, and then re-suspension was done within a 500 *μ*L binding buffer. After that, Annexin V-FITC (5 *μ*L) and PI (10 *μ*L) were utilized to treat PANC-1 cells in the ark for 15 min. At last, a flow cytometer (BD Biosciences, USA) was utilized to determine apoptotic cells [24].

### 2.7. Wound Healing Assay

Cells (2 × 10^5^ cells/well) were seeded into a 12-well culture plate and cultured in DMEM supplemented with 10% FBS. PANC-1 cells from different groups reached 100% confluence, and 200 *μ*L pipette tip was used to scratch a wound. An inverted microscope (Olympus, Japan) was adopted to observe cell images at 0 and 48 h (200×) [24].

### 2.8. Transwell Assay

PANC-1 cells were cultured into the upper compartment with the basal medium in the migration assay. Lower chambers were supplied with 600 *μ*L RPMI-1640 medium containing 10% FBS. After 48 h, cells were immobilized with methanol and stained with 0.1% crystal violet and observed under a microscope (Leica, Germany) and counted. In invasion assay, the used membrane was precoated with Matrigel (Franklin Lakes, NJ, USA). The other methods were like cell migration [24].

### 2.9. Bioinformatic Analysis for Possible MicroRNA Target Genes

We used Internet databases to predict the target gene of miR-451a, including ENCORI (http://starbase.sysu.edu.cn/), miRWalk (http://www.umm.uniheidelberg.de/apps/zmf/mirwalk), and miRDB (http://www.mirdb.org/miRDB).

### 2.10. qRT-PCR

After the total RNA extraction, cDNA was prepared with the extracted total RNA by the RNeasy plus micro kit through reverse transcription with specific instructions. The starting material of qRT-PCR was carried out using the StepOne system (Life Technologies Corp). Sequences of all primers were designed by Primer Premier software 4.0 (Premier, Canada), which are shown in Table 1. U6 or *β*-actin was normalized by the 2^−ΔΔCT^ approach [25].

### 2.11. Luciferase Reporter Assays

This study subcloned MEF2D wild-type (WT) or mutant (MT) to the pmirGLO dual-luciferase vectors (Promega, USA) for generating pmirGLO-MEF2D WT/MUT to co-transfect into PANC-1 cells with NC mimics or miR-451a mimic. Luciferase activity was detected using the Dual-Luciferase Reporter Assay System (Promega, Madison, WI, USA) at 48 h. Renilla luciferase activity was used as the endogenous control [24].

### 2.12. Oxygen Consumption

In brief, PANC-1 cells from different groups were washed by PBS and added with 150 *μ*L fresh medium. Next, 10 *μ*L R01 oxygen fluorescent probe was added and thoroughly mixed, and 100 *μ*l oxygen sealing solution was added per well. Finally, the Seahorse XFe174 96 flux analyzer (Agilent, Santa Clara, CA) detected O_2_ consumption.

### 2.13. Mitochondrial Membrane Potential Analysis

PANC-1 cells from different groups were treated with JC-1 (0.5 mL) (Yeasen, Shanghai, China) for 15 min at 37 °C. Then, the cells were centrifuged at 400 rpm/min for 5 min followed by resuspension with 2 mL buffer solution. After centrifuging at 400 rpm/min for 5 min, the cells were resuspended with 0.3 mL buffer solution. Finally, images were collected using a fluorescent microscope (Olympus, Tokyo, Japan).

### 2.14. Detection of Reactive Oxygen Species (ROS) Generation

Dichlorodihydrofluorescein diacetate (DCFH-DA) was adopted to evaluate ROS generation. Briefly, PANC-1 cells from different groups were incubated with 10 mM DCF-DA at 37°C for 15 min. After being washed three times with PBS, fluorescent microscopic images were observed under a fluorescent microscope (Olympus, Tokyo, Japan).

### 2.15. Sphere Formation Assay

PANC-1 cells (2 × 10^3^) were inoculated in low-adherent 6-well plates and cultured under serum-free conditions in DMEM-F12 containing B27 (20 *μ*L/mL), EGF (20 ng/mL), bFGF (20 ng/mL), and 1% penicillin-streptomycin for 10–12 days, and cell spheroids were counted under a microscope (Olympus, Japan).

### 2.16. Statistical Analysis

Data were analyzed using GraphPad Prism 5.0 and presented in mean ± SD. All experiments were repeated at least 3 times. Unpaired Student's *t*‐test was used to compare the differences between two independent groups. The differences between multiple groups were compared by ANOVA and Tukey's post hoc analysis. *P* < 0.05 stood for statistical significance.

## 3. Results

### 3.1. Tumor Acidic Microenvironment Promotes the Proliferation, Migration, and Invasion of PC Cells

**Figure 1:**
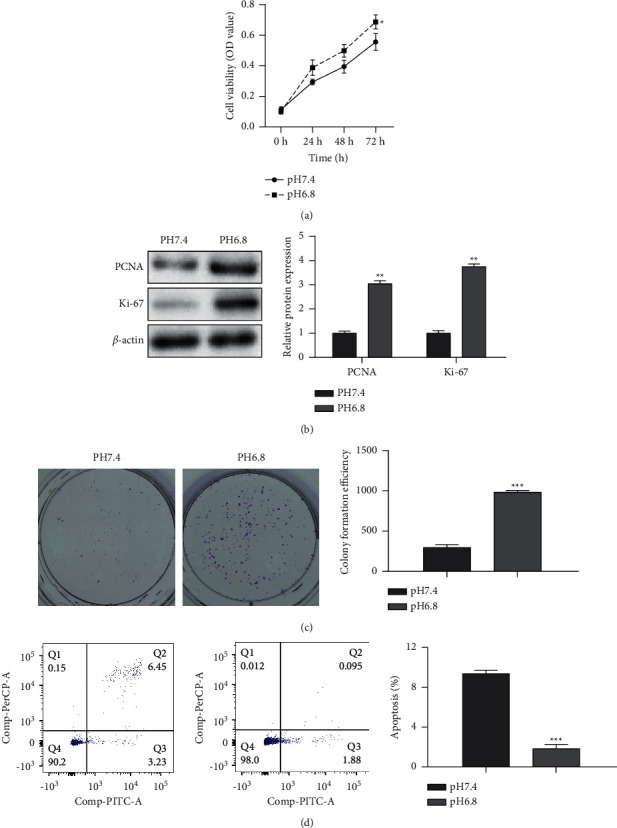
Tumor acidic microenvironment promotes the proliferation of pancreatic cancer cells. PANC-1 cells were incubated under normal (pH 7.4) and acidic conditions (pH 6.8) up to 48 h at 5% CO_2_. (a) The cell viability detected by CCK-8 assay. (b) The expression of PCNA and Ki-67 measured by western blot. (c) The clone formation assay. (d) The apoptosis detected by flow cytometry assay. Error bars, mean ± SD from three independent experiments. ^*∗∗*^*P* < 0.01; ^*∗∗∗*^*P* < 0.001.

**Figure 2:**
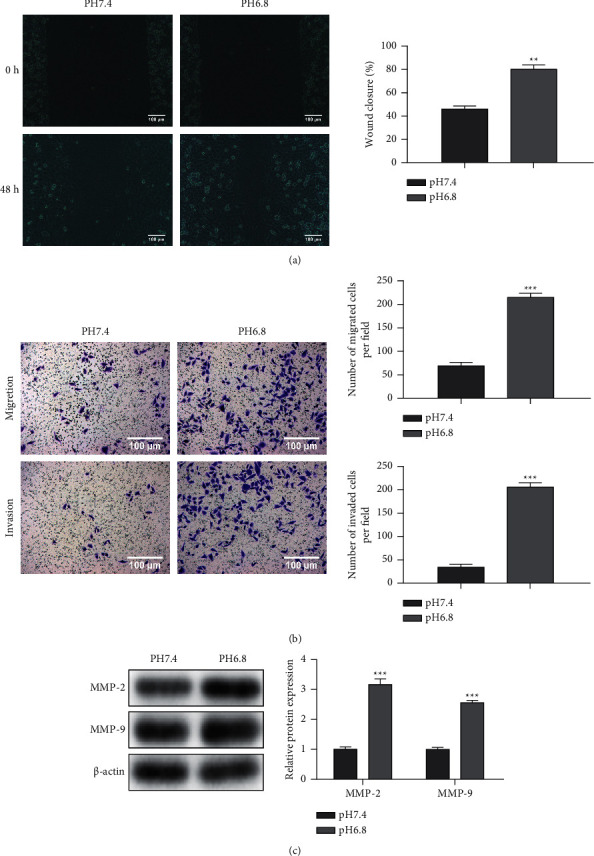
Tumor acidic microenvironment accelerates the migration and invasion of pancreatic cancer cells. (a) Cell motility detected by wound healing assay. (b) Cell motility and invasion ability assessed by transwell assay. (c) The expression of MMP-2 and MMP-9 measured by western blot. Error bars, mean ± SD from three independent experiments. ^*∗∗*^*P* < 0.01; ^*∗∗∗*^*P* < 0.001.

In order to detect whether the acidic microenvironment potentiates PC progression, PANC-1 cells were treated under normal (pH 7.4) and acidic conditions (pH 6.8) up to 48 h at 5% CO_2_. PANC-1 cells exposed to acidic conditions showed higher cell viability (Figure 1(a)). Meanwhile, the acid-treated PANC-1 cells presented increased proliferation-related proteins (PCNA and Ki-67) (Figure 1(b)), as well as significantly enhanced colony formation ability (Figure 1(c)) and reduced apoptotic cells (Figure 1(d)). In addition, wound healing and transwell assays illustrated that cell motility and invasion ability were markedly enhanced in acid-treated PANC-1 cells (Figures 2(a) and 2(b)). Also, MMP-2 and MMP-9 protein levels related to migration and invasion were significantly increased in acid-treated PANC-1 cells (Figure 2(c)). These data illustrated that an acidic microenvironment could promote the development of PC.

### 3.2. Tumor Acidic Microenvironment Downregulated the Expression of Mitochondrial Oxidative Respiration-Related Gene miR-451a

**Figure 3:**
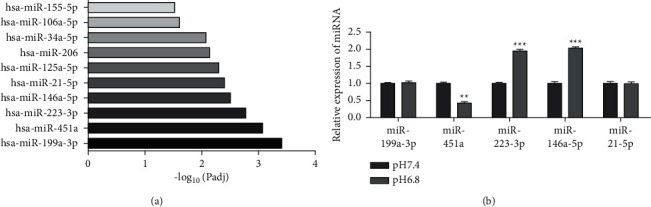
Tumor acidic microenvironment downregulated the expression of mitochondrial oxidative respiration-related gene miR-451a. (a) The functional enrichment analysis by g:Profiler software. (b) The expression of miR-199a-3p, miR-451a, miR-223-3p, miR-146-5p, and miR-21-5p detected by qRT-PCR. Error bars, mean ± SD from three independent experiments. ^*∗∗*^*P* < 0.01; ^*∗∗∗*^*P* < 0.001.

To explore whether the acidic environment promotes PC development by regulating ROS-related genes, we searched ROS-related genes through GeneCards software. We performed a functional enrichment analysis of ROS-related genes through g:Profiler software (Figure 3(a)). In the meantime, qRT-PCR analysis was conducted for assessing miR-451a expressions in PANC-1 exposed to an acidic environment.  Figure 3(b) demonstrates that, among the top five genes, only the expression of miR-451a (mitochondrial oxidative respiration-related gene) was significantly downregulated under acidic conditions.

### 3.3. MEF2D Is the miR-451a Direct Target

**Figure 4:**
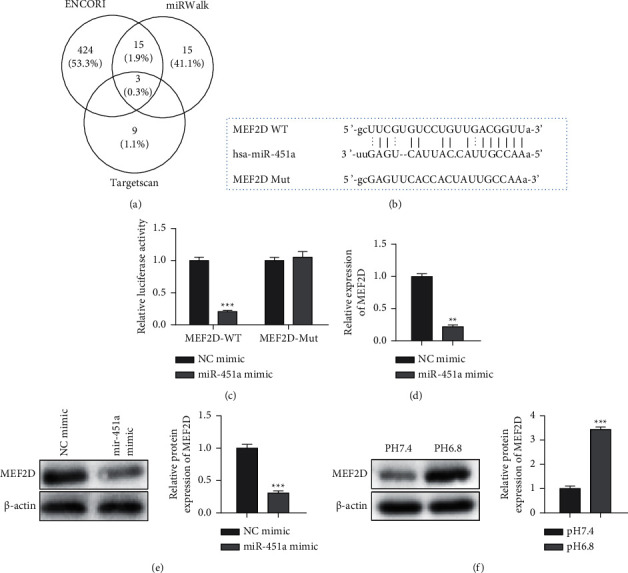
MEF2D is a target gene of miR-451a. (a) The target genes were predicted by ENCORI, miRWalk, and TargetScan. (b) The miR-451a putative binding sites and corresponding mutant sites of MEF2D. (c) Luciferase reported assay. (d) The mRNA expression of MEF2D detected by qRT-PCR in miR-451a mimics-treated PANC-1 cells. (e) The protein expression of MEF2D in miR-451a mimics-treated PANC-1 cells. (f) The protein expression of MEF2D in PANC-1 cells exposed to acidic conditions. Error bars, mean ± SD from three independent experiments. ^*∗∗*^*P* < 0.01; ^*∗∗∗*^*P* < 0.001.

To ascertain the possible miR-451a targets, bioinformatics tools were jointly utilized, and MEF2D (related to ROS) was chosen for further study (Figure 4(a)). Moreover, dual-luciferase reporter analysis further validated the association of miR-451a and MEF2D (Figures 4(b) and 4(c)). Besides, miR-451a mimics in PANC-1 cells decreased MEF2D mRNA and protein levels (Figures 4(d) and 4(e)). Moreover, MEF2D expressions were increased within PANC-1 cells exposed to acidic conditions (Figure 4(f)). Together, these data illustrated that MEF2D was a direct downstream target of miR-451a.

### 3.4. Knockdown of MEF2D Enhances MMP Loss and ROS Generation and Inhibits the Stemness of PANC-1 Cells

**Figure 5:**
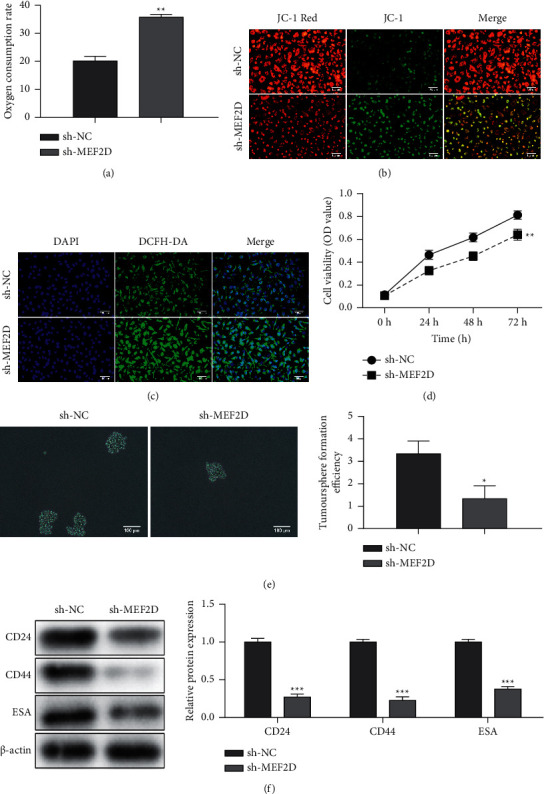
Knockdown of MEF2D enhances MMP loss and ROS generation and inhibits stemness of PANC-1 cells. PANC-1 cells were transfected with sh-NC and sh-MEF2D for 48 h. (a) The oxygen consumption rate. (b) JC-1 staining was used to detect MMP loss of PANC-1 cells. (c) DCFH staining was used to detect ROS generation of PANC-1 cells. (d) The cell viability detected by CCK-8 assay. (e) Sphere formation assay was performed. (f) The expression of CD24, CD44, and ESA. Error bars, mean ± SD from three independent experiments. ^*∗*^*P* < 0.05, ^*∗∗*^*P* < 0.01, and ^*∗∗∗*^*P* < 0.001.

To verify whether MEF2D regulated the progression of PC by affecting mitochondrial function, sh-MEF2D was transfected into PANC-1 cells for 48 h, and the mitochondrial bioenergetics profile was recorded. Knockdown of MEF2D increased the oxygen consumption in PANC-1 cells (Figure 5(a)). Moreover, MMP loss played a central role in apoptosis, and ROS contributed to MMP loss. Therefore, we detected the effect of MEF2D on the mitochondrial membrane potential by JC-1 analysis, in which red fluorescence represented the mitochondrial aggregate JC-1 in normal mitochondria and green fluorescence indicated the monomeric JC-1 in unhealthy mitochondria.  Figure 5(b) demonstrates that MEF2D knockdown promoted the MMP loss in PANC-1 cells (Figure 5(b)). Besides, MEF2D downregulation upregulated the ROS generation in PANC-1 cells (Figure 5(c)). Additionally, the silence of MEF2D suppressed the PANC-1 cell viability (Figure 5(d)). Sphere formation and western blot assays were used to analyze cell stemness. The data suggested that knockdown of MEF2D significantly inhibited sphere formation and the expression of CD24, CD44, and ESA (stemness markers) (Figures 5(e), 5(f)).

### 3.5. Tumor Acidic Microenvironment Regulates the Function of Mitochondria via the miR-451a/MEF2D Axis

**Figure 6:**
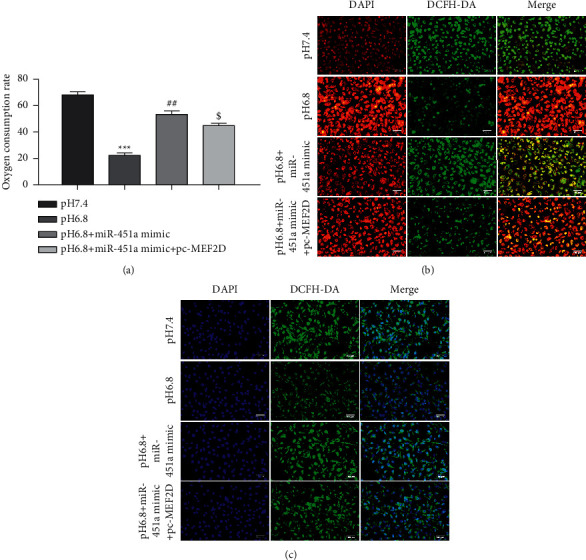
Tumor acidic microenvironment regulates the function of mitochondria via miR-451a/MEF2D axis. Under acidic conditions, PANC-1 cells were transfected with miR-451a mimics, pc-MEF2D, and the corresponding control group. (a) The oxygen consumption rate. (b) JC-1 staining was used to detect MMP loss of PANC-1 cells. (c) DCFH staining was used to detect ROS generation of PANC-1 cells. Error bars, mean ± SD from three independent experiments. ^$^*P* < 0.05, ^##^*P* < 0.01, and ^*∗∗∗*^*P* < 0.001.

The results above further investigated whether the acidic environment affected mitochondrial function by modulating the miR-451a/MEF2D axis in PANC-1 cells. As shown in Figure 6(a), miR-451a overexpression inhibited the decrease of oxygen consumption caused by acidic conditions, while MEF2D upregulation partially restored the effects of miR-451a mimics on the oxygen consumption. Moreover, miR-451a mimics promoted the MMP loss and ROS generation in PANC-1 cells under acidic conditions, partially rescued by MEF2D upregulation (Figures 6(b), 6(c)).

### 3.6. Tumor Acidic Microenvironment Modulates the Proliferation, Migration, and Stem Cell Stemness of PC through the miR-451a/MEF2D Axis

**Figure 7:**
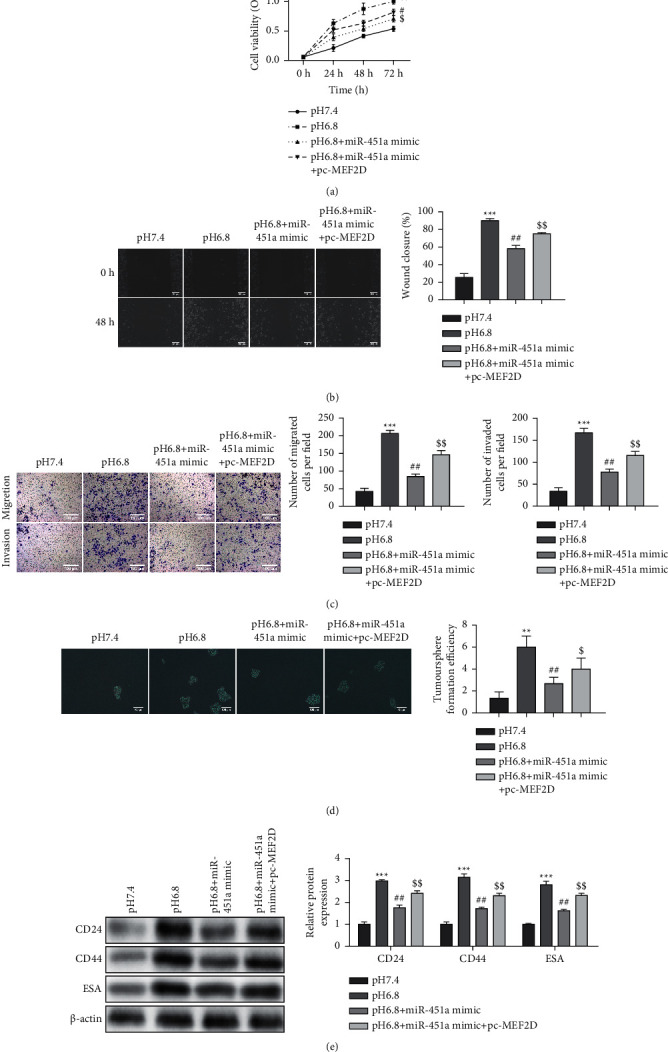
Tumor acidic microenvironment modulates the proliferation, migration, and stem cell stemness of PC through the miR-451a/MEF2D axis. Under acidic conditions, PANC-1 cells were transfected with miR-451a mimics, pc-MEF2D, and the corresponding control group. (a) The cell viability detected by CCK-8 assay. (b) Cell motility detected by wound healing assay. (c) Cell motility and invasion ability assessed by transwell assay. (d) Sphere formation assay was performed. (e) The expression of CD24, CD44, and ESA. Error bars, mean ± SD from three independent experiments. ^*∗∗*^*P* < 0.01, ^*∗∗∗*^*P* < 0.001, ^#^*P* < 0.05, ^##^*P* < 0.01, ^$^*P* < 0.05, and ^$$^*P* < 0.01.

Finally, we further verified our hypothesis that an acidic environment modulated PC's proliferation, migration, and stem cell stemness through the miR-451a/MEF2D axis. CCK-8, wound, healing, and transwell analyses revealed that MEF2D overexpression partly reversed the reduction of functional cell capabilities caused by miR-451a mimics in an acidic environment (Figures 7(a)–7(c)). Furthermore, miR-451a upregulation significantly inhibited the increase of sphere formation and the increase of CD24, CD44, and ESA expression caused by the acidic conditions, while MEF2D overexpression reversed the functions of miR-451a mimics (Figures 7(d), 7(e)).

## 4. Discussion

This study discovered that the acidic microenvironment promoted cell proliferation, migration, and invasion and reduced apoptosis in PC. Importantly, our results found that the acidic microenvironment decreased miR-451a expression, related to mitochondrial oxidative respiration, which enhanced the oxygen consumption, MMP loss, and ROS generation and inhibited the proliferation, migration, invasion, and stem cell stemness of PC cells under acidic conditions. In addition, we predicted and confirmed a novel target of miR-451a, MEF2D, representing an oncogenic protein that could regulate tumor suppression in PC. Like miR-451a mimics, knockdown of MEF2D also increased the oxygen consumption, MMP loss, and ROS generation with a consequent increase of apoptosis. Furthermore, MEF2D overexpression reversed the effects of miR-451a mimics on the functional effects of PC. To the best of our knowledge, our study firstly demonstrated that the acidic microenvironment accelerated PC progression by modulating the miR-451a/MEF2D pathway.

The acidic tumor microenvironment is caused by the accumulation of acidic metabolites in the tumor microenvironment due to enhanced glycolysis, hypoxia, and tissue insufficiency [26]. Increasing studies have shown that the acidic microenvironment exerted essential effects on tumor proliferation, invasion, and migration and immune escape and affects the effective treatment of tumors, making tumor cells insensitive to radiotherapy and chemotherapy, which makes tumor treatment more difficult [27, 28]. For example, under an acidic environment, BRAF^v600E^ melanoma cells manifested active proliferation, enhanced antiapoptotic ability, and exhibited more resistance to the BRAF inhibitor vemurafenib [29]. Noteworthy, it has been found that the acidic microenvironment induces epithelial-mesenchymal transition in PC cells [18] and thus affects the migration and invasion of cancer cells. Analogously, in our study, acidic conditions increased PANC-1 cell proliferation, migration, and invasion.

MiRNAs have been confirmed to participate in post-transcriptional gene expression regulation, acting as oncogenes or tumor suppressors [30, 31]. In addition, the evidence demonstrated that tumor cells exhibit increased intrinsic ROS stress due to increased metabolic activity and mitochondrial malfunction [32]. In this study, we focused on the effects of miRNAs on the functional activities of PC. Through bioinformatics analysis, miR-451a (related to ROS) expressions were downregulated in PANC-1 cells under acidic conditions. miR-451a has been reported to have antineoplastic effects on multiple cancers, including lung cancer [33], papillary thyroid cancer [34], colorectal cancer [35], and breast cancer. Our study found that miR-451a upregulation reversed the effects of acid conditions on oxygen consumption, MMP loss, and ROS generation in PC cells, as well as the proliferation, migration, invasion, and stem cell stemness. The above results suggested that the acidic tumor microenvironment played a crucial role in PC progression, inhibiting miR-451a.

MEF2D, a member of the MEF2 family, is a crucial developmental factor, showing an essential role in the tumorigenesis of various malignant tumor cells. Firstly, it was reported in the experimental study of leukemia that MEF2D promoted the malignant transformation of normal cells [36]. Subsequent evidence showed that MEF2D exerted significant roles in the progression of hepatocellular carcinoma, colorectal cancer, lung cancer, and osteosarcoma [37–40]. Song et al. found that MEF2D controlled cell proliferation, migration, and invasion abilities in pancreatic cancer via the Akt/GSK-3*β* signaling pathway [41]. Our data illustrated that MEF2D, a target gene of miR-451a, was highly expressed under acidic conditions. The silence of MEF2D increased the oxygen consumption, MMP loss, and ROS generation with a consequent increase in apoptosis. In addition, MEF2D overexpression reversed the effects of miR-451a mimics on the functional activities of PC, which was consistent with previous reports [41].

In summary, we reported a novel molecular signaling pathway that the acidic tumor microenvironment promotes PC progression through the miR-451a/MEF2D axis, which provides a novel therapeutic target for PC.

## Figures and Tables

**Table 1:** Primer sequences used for qRT-PCR.

Genes		Primer sequences (5′-3′)
MEF2D	Forward	AGGGAAATAACCAAAAAACTACCAAA
	Reverse	GCTACATGAACACAAAAACAGAGACC

miR-199a-3p	Forward	ACACTCCAGCTGGGACAGTAGTCTGCACAT
	Reverse	CTCAACTGGTGTCGTGGAGTCGGCAATTCAGTTGAGTAACCAAT

miR-451a	Forward	ACCGTTACCATTACT
	Reverse	CTCACACGACTCACGA

miR-223-3p	Forward	GCCGAGACCCCAUAAACUG
	Reverse	CAGTGCGTGTCGTGGAGT

miR-146a-5p	Forward	UGAGAACUGAAUUCCAUGGGUU
	Reverse	ACTCTTGACTTAAGGTACCCAA

miR-21-5p	Forward	GCTTATCAGACTGATGTTG
	Reverse	GAACATGTCTGCGTATCTC

*β*-actin	Forward	CTCGCCTTTGCCGATCC
	Reverse	GGGGTACTTCAGGGTGAGGA

U6	Forward	CTCGCTTCGGCAGCACA
	Reverse	AACGCTTCACGAATTTGCGT

## Data Availability

All data generated or analyzed during this study are included within this article.
